# Repeated Administration of the Cannabinoid WIN Alters the Isoflurane-Sparing Effect of Morphine and Dexmedetomidine

**DOI:** 10.3390/vetsci10050310

**Published:** 2023-04-24

**Authors:** José Antonio Ibancovichi, Julio Raúl Chávez-Monteagudo, Pedro Sánchez-Aparicio, Marco Antonio De Paz-Campos

**Affiliations:** 1 Departamento de Anestesiología y Analgesia, Facultad de Medicina Veterinaria, Universidad Autónoma del Estado de México, UAEM, Toluca 50000, Mexico; 2 Departamento de Ciencias Pecuarias, Facultad de Estudios Superiores Cuautitlán, Hospital de Pequeñas Especies, Universidad Nacional Autónoma de México, UNAM, Cuautitlán Izcalli 54740, Mexico; 3 Departamento de Farmacología, Facultad de Medicina Veterinaria, Universidad Autónoma del Estado de México, UAEM, Toluca 50000, Mexico; 4 Departamento de Ciencias Biológicas, Facultad de Estudios Superiores Cuautitlán, Hospital de Pequeñas Especies, Universidad Nacional Autónoma de México, UNAM, Cuautitlán Izcalli 54740, Mexico

**Keywords:** minimum alveolar concentration of isoflurane, dexmedetomidine, morphine, cannabinoid WIN 55,212-2, rats

## Abstract

**Simple Summary:**

We believe it is essential to understand the effects of continuous cannabinoid administration on the requirements of inhalation anesthetics for patients administered general anesthesia and those administered morphine and dexmedetomidine. To understand the interaction of isoflurane with morphine and dexmedetomidine in rats treated repeatedly with the synthetic cannabinoid WIN 55,212-2, we determined the minimum alveolar concentration of isoflurane in 32 rats. We found that, in rats constantly medicated with the cannabinoid, the sparing effect of morphine on isoflurane decreased and the sparing effect of dexmedetomidine on isoflurane increased.

**Abstract:**

The impacts of morphine and dexmedetomidine on the MAC of isoflurane were studied in rats constantly medicated with the cannabinoid WIN 55,212-2. Methods: Prior to the administration of morphine, the MAC was measured in both untreated rats (MAC _(ISO)_) and those treated with a cannabinoid (MAC _(ISO + CANN)_). The effects of morphine (MAC _(ISO + MOR)_) and dexmedetomidine (MAC _(ISO + DEX)_) on untreated rats and rats treated for 21 days with the cannabinoids (MAC _(ISO + CANN + MOR)_) and (MAC _(ISO + CANN + DEX_) were also studied. Results: MAC _(ISO)_ was 1.32 ± 0.06, and MAC _(ISO + CANN)_ was 1.69 ± 0.09. MAC _(ISO + MOR)_ was 0.97 ± 0.02 (26% less than MAC _(ISO)_). MAC _(ISO + CANN + MOR)_ was 1.55 ± 0.08 (8% less than MAC _(ISO + CANN)_), MAC _(ISO + DEX)_ was 0.68 ± 0.10 (48% less than MAC _(ISO)_), and MAC _(ISO + CANN + DEX)_ was 0.67 ± 0.08 (60% less than MAC _(ISO + CANN)_). Conclusions: Medication with a cannabinoid for 21 days augmented the MAC of isoflurane. The sparing effect of morphine on isoflurane is lower in rats constantly medicated with a cannabinoid. The sparing effect of dexmedetomidine on the minimum alveolar concentration of isoflurane is greater in rats repeatedly medicated with a cannabinoid.

## 1. Introduction

There are many unresolved questions on cannabinoids. In addition, information on cannabinoids is lacking in terms of scientific evidence. In the year 2020, about 209 million people used cannabis (marihuana). *Cannabis sativa* is the most popular illicit drug of the 21st century (according to the World Drug Report 2022, published by the United Nations [1], cannabis remains the most widely used drug in the world), and countries are increasingly legalizing its medicinal and recreational use. The identification and isolation of the CB1 and CB2 cannabinoid receptors and the identification of endogenous cannabinoids have led to the assumption that the endocannabinoid system may be involved in various disease processes. Therefore, these receptors are widely studied for their possible health benefits and involvement in different diseases. In the future, cannabis and its derivatives are expected to be used for medicinal purposes in treating chronic pain (particularly in cancer) and pediatric seizure disorders, stimulating appetite, treating spasticity in multiple sclerosis, controlling nausea and vomiting in patients with HIV/AIDS, treating neurodegenerative diseases, and treating post-traumatic stress disorder and addictions to different substances. Multiple investigations are underway on the use of cannabis for treating various human diseases [2,3,4]. Given the excessive use of opioids, there is evidence that cannabinoids can help reduce opioid consumption (by up to 64% [5]) by allowing the opioid dose to be reduced by potentiating its analgesic effect [6]. Similarly, there is evidence that the joint administration of cannabinoids and opioids can reduce the perception of pain by up to 27% without generating alterations in the plasma concentration of opioids and without altering their pharmacokinetic characteristics [7].

Dexmedetomidine administration during general anesthesia procedures decreases opioid and hypnotic requirements (sparing effect); has an analgesic effect; decreases postoperative nausea, vomiting, and shivering [8]; exerts therapeutic effects with respect to perioperative stress and postoperative delirium, enabling smoother recovery; and provides neuroprotection Even though these positive effects have been demonstrated in the patient, there are studies that have focused on elucidating these effects. However, the physiological mechanisms that explain these effects are still not entirely clear [9,10,11].

Currently, animals with chronic pain can be medicated with cannabis sativa or its derivatives. If one of these patients requires general anesthesia, the interaction of cannabis derivatives with anesthetic agents is unknown. We consider it important to study whether the sparing effects of morphine and dexmedetomidine, previously reported in the literature, are the same in medicated cannabis patients. We hypothesize that the isoflurane-sparing effects of morphine and dexmedetomidine are different in rats that are repeatedly administered a synthetic cannabinoid.

## 2. Materials and Methods

This investigation and its procedures (protocol 3492/2013CHT) were approved by the Facultad de Medicina Veterinaria of the Universidad Autónoma del Estado de México.

In total, 32 rats weighing 310 ± 20 g each were placed in Plexiglas cages at a temperature of 22 ± 3 °C. The rats were offered food and water ad libitum (Prolab1 RMH 2500, St. Louis, MO, USA). The number of animals included in the study was determined according to the methodology described by Charan and Biswas [12].

The rats were handled according to international recommendations for animal studies [13].

### 2.1. Anesthetic Procedure

For anesthetization, each rat was placed in an induction chamber and administered 5% isoflurane vaporized in 100% oxygen (Forane; Baxter Laboratories, Irvine, CA, USA). The flow was 5 L/min.

Once a rat was anesthetized (verified via observation), it was removed from the chamber and placed in a supine position. Next, intubation was carried out. The larynx was visualized with a laryngoscope and, with the use of a flexible wire as a guide, a 16 G Teflon catheter (Introcan; B-Braun, Sao Goncalo, Brazil) secured to the maxilla was placed inside the trachea. To ensure that the catheter was placed properly, we used a CO_2_ analyzer (BeneView T5, Mindray, Multi-Gas Offers, Nanshan, China) connected to an anesthetic circuit, with a T-piece through which we provided fresh gas flow at 1 L/min. The amount of isoflurane was adjusted according to the effect on the heart rate and blood pressure and the presence of the eyelid reflex during instrumentation. Animals always breathed spontaneously.

To register continuous systolic (SAP), diastolic (DAP), and mean (MAP) blood pressure and heart rate (HR), we dissected the carotid artery and placed a 24 G catheter (Introcan; B-Braun, São Gonçalo, Brazil) connected to a pressure transducer (BeneView T5, Mindray, Nanshan, Nanshan, China). Before beginning each experiment, the transducer was zero calibrated, exposing the transducer to atmospheric pressure and placing it at heart level. Through this catheter, we collected 0.3 mL of arterial blood gases (GEM Premier 3000; Instrumentation Laboratory, Seattle, WA, USA) for analysis at the time of MAC determination to guarantee that the physiological parameters were within normal ranges. A catheter was also placed in the caudal vein to administer morphine and dexmedetomidine.

Through the catheter previously placed in the trachea, we collected an endotracheal gas sample and used a gas analyzer (BeneView T5, Mindray, China) to continuously measure the inspired isoflurane (FiIso), the end-tidal (FeIso), PEtCO_2_ (end-tidal carbon dioxide tension), and the respiratory rate (RR). The body temperature was maintained between 37 and 38 °C using a constant hot air circulation heating system (Equator1, SurgiVet1, Smiths Medical PM Inc., San Clemente, CA, USA).

### 2.2. Minimum Alveolar Concentration Determination

According to what was previously reported [14], we adjusted the FeIso to 1.3% and began to determine the minimum alveolar concentration (MAC). This was maintained for 15 min to allow equilibrium to be established between the alveolar gas, arterial blood, and the spinal cord [15]. Minimum alveolar concentration of isoflurane was obtained using the tail clamp method [16]. A supramaximal painful noxious stimulus was applied with a hemostat clamped (8-inch Rochester Dean) on the tail at a specific end-tidal concentration. The tail was clamped to the first ratchet lock, and the stimulus applied to the tail lasted 60 s and stopped if it elicited a positive response. A response was considered positive if the rat shook or moved its head, trunk, pelvic limbs, or thoracic limbs. A response was considered negative if the rat moved its tail, if muscle rigidity was observed, if the rat swallowed, if it chewed, or if no movement was observed. On the tail, stimuli were always applied in places other than the initial site.

If we observed a negative response, we decreased the isoflurane concentration by 10%; maintained this level for 15 min, waiting for equilibrium to be established between alveolar gas, arterial blood, and the central nervous system; and applied the stimulus again. If a positive response was observed, we increased the isoflurane concentration by 10%; maintained this level for 15 min, waiting for equilibrium to be established between alveolar gas, arterial blood, and the central nervous system; and applied the stimulus. The person who evaluated the MAC (who applied supramaximal stimulus and observed if the response was positive or negative) performed it twice on each rat and was unaware of the applied treatment at all times.

The present study was carried out at an elevation of 2680 m above sea level with an average pressure of 556 mmHg. Therefore, we corrected the minimum alveolar concentration values to sea level by applying the following formula: (barometric pressure of the location/760 mmHg) × obtained MAC value.

At the end of the experiment, we increased the concentration of isoflurane in each rat to 5%. We waited for 15 min for the concentration to reach equilibrium, and then administered sodium pentobarbital intravenously to euthanize the rats (Anestesal, Pfizer, Toluca, Mexico).

### 2.3. Experimental Design

The rats were randomly assigned to four groups (*n* = 8) using Excel 2007 (Microsoft Office).

For the MAC _(ISO + MOR)_ group, we performed the measurement 45 min after the administration of 3 mg/kg of morphine i.v. (Graten, PiSA, Mexico). This is the optimal dose as determined in a previous publication [17]. The MAC of isoflurane (MAC _(ISO)_) was measured before morphine administration; this was considered as the control group, and was used for the comparison of the rest of the treatments.

For the MAC _(ISO + DEX)_ group, the measurement was performed 30 min after the administration of a continuous intravenous infusion of 0.25 μg/kg/min of dexmedetomidine i.v. (Dexdomitor, Zoetis, Mexico). This is the dose determined to be optimal in a previous publication [18]. 

For the MAC _(ISO + CANN + MOR)_ and the MAC _(ISO + CANN + DEX)_ groups, we intraperitoneally (i.p.) administered 1 mg/kg of WIN (mesylate salt, Sigma–Aldrich, St. Louis, MO, USA) every 24 h for 21 days, in accordance with Lawston et al. [19]. For the MAC _(ISO + CANN + MOR)_ group, the measurement was performed 24 h after the last treatment (day 21). Then, 45 min prior to MAC measurement, 3 mg/kg of morphine was administered i.v. The MAC of isoflurane (MAC _(ISO + CANN)_) was measured before morphine administration, while, for the MAC _(ISO + CANN + DEX)_ group, the measurement was performed 24 h after the last treatment (day 22), 30 min after the continuous intravenous infusion of 0.25 μg/kg/min of dexmedetomidine i.v.

WIN was prepared in Tween 80 according to Tanda et al. [20]. A 0.9% saline solution was used to dilute a 0.3% Tween solution.

### 2.4. Statistical Analysis

We used the statistical program Prism 6 (GraphPad Software, Inc., San Diego, CA, USA). We carried out a Shapiro–Wilk test to determine data normality and a Holm–Sidak test to perform analysis of variance and for post hoc testing between groups. A statistical difference was considered at *p* < 0.05. Data are reported as mean ± standard deviation (SD). 

## 3. Results

**Figure 1:**
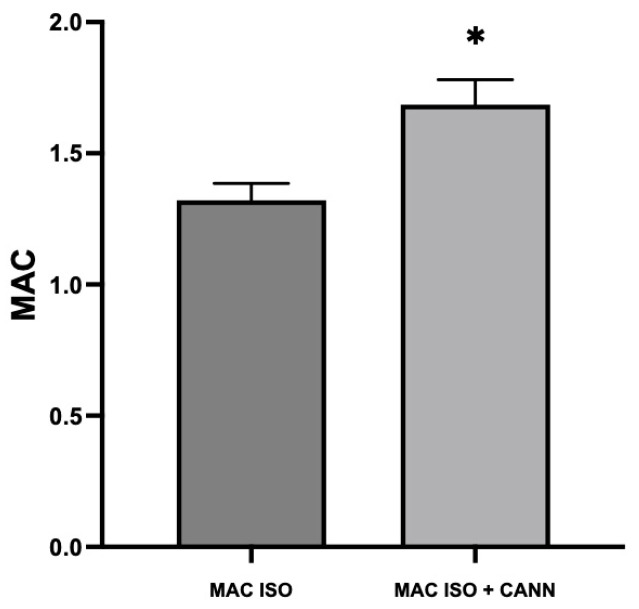
Effect of the cannabinoid WIN on the MAC of isoflurane. MAC _(ISO)_ was 1.32 ± 0.06 and 1.69 ± 0.09 in the MAC _(ISO + CANN)_ group. An increase in the minimum alveolar concentration was observed. [*] Statistically significant compared with the control group *p* < 0.0001.

**Figure 2:**
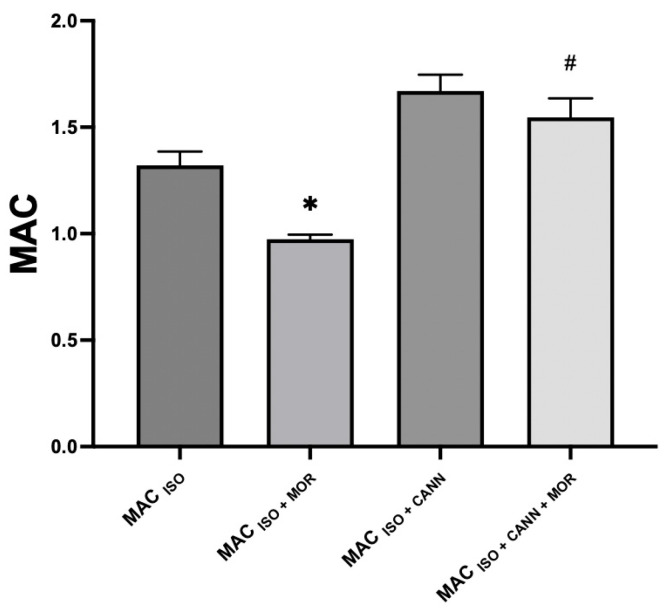
Sparing effect of morphine in unmedicated rats and rats medicated for 21 days with the cannabinoid WIN. MAC _(ISO + MOR)_ was 0.97 ± 0.02 (26% less than MAC _(ISO)_). MAC _(ISO + CANN + MOR)_ was 1.55 ± 0.08 (8% less than MAC _(ISO + CANN)_). The sparing effect of morphine on isoflurane was lower in rats constantly medicated with a cannabinoid compared with the effect of morphine in rats not treated with the cannabinoid. [*] Statistically significant between MAC _ISO + MOR_ and the MAC _ISO_ group *p* < 0.0001. (#) Statistically significant between MAC _ISO + CANN + MOR_ and MAC _ISO + CANN_ group *p =* 0.0094.

**Figure 3:**
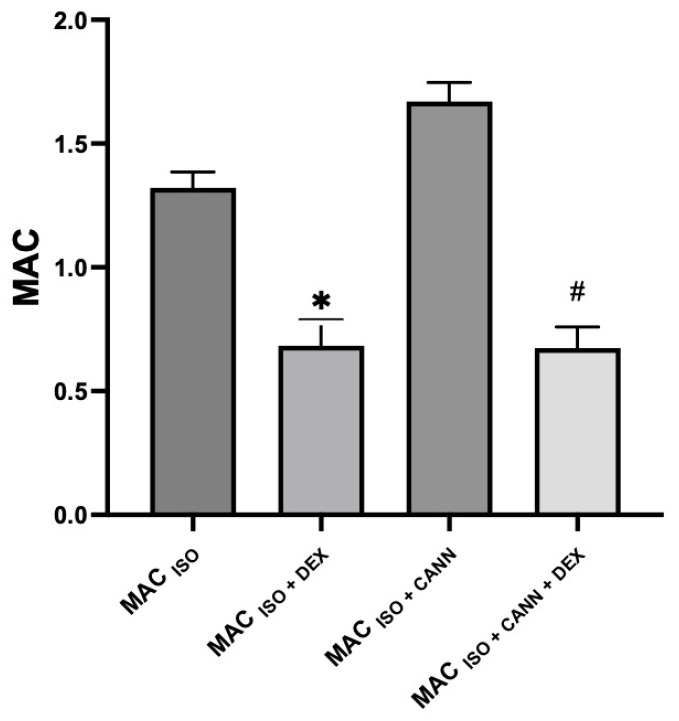
Sparing effect of dexmedetomidine in unmedicated rats and rats medicated for 21 days with the cannabinoid WIN. MAC _(ISO + DEX)_ was 0.68 ± 0.10 (48% less than MAC _(ISO)_). MAC _(ISO + CANN + DEX)_ was 0.67 ± 0.08 (60% less than MAC _(ISO + CANN)_). The sparing effect of dexmedetomidine on the minimum alveolar concentration of isoflurane was greater in rats repeatedly medicated with a cannabinoid compared with the effect of dexmedetomidine in rats not treated with the cannabinoid. [*] Statistically significant between the MAC _Iso + Dex_ and the MAC _ISO_ group *p* < 0.0001. (#) Statistically significant between MAC _ISO + CANN + DEX_ and the MAC _ISO + CANN_ group *p* < 0.0001.

MAC _(ISO)_ was 1.32 ± 0.06 and MAC _(ISO + CANN)_ was 1.69 ± 0.09, in agreement with the results of previously published studies [21]. MAC _(ISO + MOR)_ was 0.97 ± 0.02 (26% less than MAC _(ISO)_). MAC _(ISO + CANN + MOR)_ was 1.55 ± 0.08 (8% less than MAC _(ISO + CANN)_), MAC _(ISO + DEX)_ was 0.68 ± 0.10 (48% less than MAC _(ISO)_), and MAC _(ISO + CANN + DEX)_ was 0.67 ± 0.08 (60% less than MAC _(ISO + CANN)_). These results were previously published as a preprint [22]. Table 1 presents the different MAC values. Figure 1 shows the increase in the MAC of isoflurane when a synthetic cannabinoid was administered for 21 days. Figure 2 shows an observed decrease in the effect of morphine on the MAC of isoflurane. In Figure 3 shows an observed increase in the sparing effect of dexmedetomidine in rats treated for 21 days with a cannabinoid. Table 2 presents the cardiorespiratory and temperature values of the different groups. There was a statistical difference in the heart rates in the groups treated with dexmedetomidine when compared with their respective control groups. However, there were no statistically significant differences between groups in terms of other variables. During morphine administration, a transient decrease in heart rate was observed.

## 4. Discussion

In this study, we observed that the repeated administration of a cannabinoid for 21 days augmented the minimum alveolar concentration of isoflurane. This result coincides with those previously reported [21], in which the increase in the minimum alveolar concentration of isoflurane was attributed to an increase in norepinephrine efflux (previously reported by Page et al. [23]) because of the repeated administration of cannabinoids. The present study was not designed to determine the causes of the observed increase in the minimum alveolar concentration of isoflurane. We do not know the minimum time necessary to generate the observed effect. It will also be important to determine whether all varieties of cannabinoids have the same effect on the minimum alveolar concentration. Similarly, it is unknown whether the cannabinoid used in the present study has the same effect on the MAC of sevoflurane and desflurane. It is also important to determine whether the chronic administration of the different cannabinoids also alters the requirements of the different available injectable anesthetic drugs. At the time the minimum alveolar concentration of isoflurane was determined, the cardiovascular parameters and temperature were within the normal range and did not present statistical differences between groups medicated with cannabinoids and morphine. According to the results, morphine decreased the MAC of isoflurane by 26%, which coincides with results previously reported in the literature [17], but the isoflurane-sparing effect of morphine was weaker in rats chronically treated with a cannabinoid (the minimum alveolar concentration of isoflurane was reduced by only 8%). This can be a consequence of increased noradrenergic activity in the central nervous system due to the chronic administration of cannabinoids [24,25]. It is also interesting to note that there are reports of a cross-tolerance effect between opioid and cannabinoid compounds. The administration of Δ9-THC has been observed to induce the tolerance of the analgesic and cardiovascular effects of morphine [26], and the chronic administration of morphine reportedly induces tolerance of the analgesic effects of Δ9-THC [27]. The mechanism by which this effect is generated is complex and remains unclear [28]. The decrease in the effect of morphine, observed as the lowering of the MAC of isoflurane in rats repeatedly medicated with a cannabinoid, could suggest (in MAC terms) a cross-tolerance effect between cannabinoids and morphine. To the best of the authors’ knowledge, it is unknown whether the different opioids available could cause the same effect.

A statistical difference was observed in the heart rates when the groups medicated with dexmedetomidine were compared with their respective controls. The dexmedetomidine generated a marked bradycardia. This effect on heart rate coincides with the already known cardiovascular effects of α2-adrenoceptor agonists [18]. With respect to the other variables, statistically significant differences were not observed between groups. We also observed that the effect of dexmedetomidine on the minimum alveolar concentration of isoflurane was constant in animals medicated in a sustained manner with a cannabinoid. At the doses used, we observed a reduction of 48% in the minimum alveolar concentration in rats without the effect of the cannabinoid and a decrease of 60% in the MAC in rats repeatedly medicated with WIN. These results indicate that the repeated use of a cannabinoid modifies the isoflurane-sparing effect of dexmedetomidine, unlike what happens with morphine, where we observed a decrease. Although the effect of dexmedetomidine on the sparing effect of isoflurane is already known [29], to the best of the authors’ knowledge, this study is the first to report the increased isoflurane-sparing effect of dexmedetomidine in rats medicated for 21 days with a cannabinoid. Dexmedetomidine, an α2-adrenoceptor agonist, is frequently used to control anxiety, produce variable degrees of sedation depending on the dose administered, generate analgesia, and assist in multimodal analgesia techniques in general anesthesia procedures and in the intensive care unit. Sympatholytic drugs decrease central sympathetic activity, the present concentrations of catecholamines [30], and the halothane MAC (by up to 90%) [31]. Therefore, dexmedetomidine lowers the requirements of inhalational anesthetics by processes other than inhibiting noradrenaline in the nervous system; it has been proven that the locus coeruleus is not the only place where α2-adrenoceptor agonists exercise their mechanisms of action [32]. It is important to determine how the constant administration of a synthetic cannabinoid favors the effect of dexmedetomidine on the requirements of inhalational anesthetics. Unfortunately, our study did not allow us to determine this mechanism. We do not know whether all available α2-adrenoceptor agonists have the same effect. Therefore, it is important to consider the possibility of modifying the anesthetic requirements of individuals who consume or are treated with cannabinoids. These patients may present different responses to the usual doses of morphine and dexmedetomidine during inhalation anesthesia. The authors think it is essential to understand the effects of continuous cannabinoid administration on the requirements of inhalation anesthetics for patients administered general anesthesia and those administered morphine and dexmedetomidine. We feel that it is important to study the various effects that the chronic consumption of cannabinoids could have on the processes involved in the anesthetic and analgesic techniques carried out in different animal species, including humans. The results of this study should be interpreted with caution since WIN is a synthetic cannabinoid and may not reflect the effect of consuming natural cannabinoids.

## 5. Conclusions

The administration of a cannabinoid for 21 days alters the minimum alveolar concentration of isoflurane. We observed that the concentration of isoflurane required to prevent movement in response to a painful stimulus is higher in individuals who were repeatedly administered cannabinoid. The sparing effect of morphine on isoflurane is lower in rats constantly medicated with a cannabinoid. Therefore, it will be prudent to consider that individuals who have been under the effect of a synthetic cannabinoid may present different effects than expected when administering morphine, since cannabinoids decrease the sparing effect of morphine compared with individuals who do not consume cannabinoids. The sparing effect of dexmedetomidine on the minimum alveolar concentration of isoflurane is greater in rats repeatedly medicated with a cannabinoid. Because of this, it will be necessary to consider reducing the dose of isoflurane in patients who are constantly exposed to cannabinoids and are administered dexmedetomidine.

## Figures and Tables

**Table 1:** Repeated administration of the cannabinoid WIN alters the isoflurane-Sparing Effects of Morphine and Dexmedetomidine.

Group	MAC%	SD	% MAC Increase (↑) or Decrease (↓)	*p*-Value	95% CI
MAC _(ISO)_	1.32	0.06		-	1.27–1.37
MAC (_ISO + CANN)_	1.69 *	0.09	↑ 28%	<0.0001	1.61–1.76
MAC _(ISO + MOR)_	0.97 *	0.02	↓ 26%	<0.0001	0.95–0.99
MAC _(ISO + CANN + MOR)_	1.55 ^+^	0.08	↓ 8%	0.0094	1.47–1.62
MAC _(ISO + DEX)_	0.68 *	0.10	↓ 48%	<0.0001	0.59–0.77
MAC _(ISO + CANN + DEX)_	0.67 ^+^	0.08	↓ 60%	<0.0001	0.60–0.74

* Statistically significant compared with the control group, MAC _(ISO)_ (*p* < 0.05). ^+^ Statistically significant compared with the control group, MAC _(ISO + CANN)_ (*p* < 0.05).

**Table 2:** Cardiorespiratory and temperature values of the different groups.

Value	MAC _(ISO)_	MAC _(ISO + CANN)_	MAC _(ISO + MOR)_	MAC _(ISO + CANN + MOR)_	MAC _(ISO + DEX)_	MAC _(ISO + CANN + DEX)_
Heart rate (bpm)	401 ± 8	403 ± 7	399 ± 8	401 ± 11	303 ± 16 *	297 ± 21 ^+^
MAP (mmHg)	93 ± 8	90 ± 9	91 ± 7	92 ± 8	86 ± 7	84 ± 11
Temperature °C	37.7 ± 0.07	37.6 ± 0.12	37.2 ± 0.11	37.4 ± 0.09	37.4 ± 0.10	37.5 ± 0.08
pH	7.3 ± 03	7.3 ± 0.04	7.4 ± 0.02	7.3 ± 0.02	7.3 ± 0.06	7.3 ± 0.09
PaO_2_ (mmHg)	301 ± 34	295 ± 8	299 ± 12	289 ± 10	291 ± 16	289 ± 13
PaCo_2_ (mmHg)	37 ± 4	37 ± 1	38 ± 2	38 ± 4	32 ± 2	37 ± 7

* Statistically significant (*p* < 0.0001) compared with the MAC _(ISO)_ group. ^+^ Statistically significant (*p* < 0.0001) compared with the MAC _(ISO + CANN)_ group.

## Data Availability

The data presented in this study are available on request from the corresponding author.
